# Chemical Composition of Methanol Extracts from Leaves and Flowers of *Anemonopsis macrophylla* (Ranunculaceae)

**DOI:** 10.3390/ijms25020989

**Published:** 2024-01-12

**Authors:** Vera A. Kostikova, Natalia V. Petrova, Alexander A. Chernonosov, Vladimir V. Koval, Evgeniia R. Kovaleva, Wei Wang, Andrey S. Erst

**Affiliations:** 1 Central Siberian Botanical Garden, Siberian Branch of Russian Academy of Sciences (SB RAS), Novosibirsk 630090, Russia; e.kovaleva2@g.nsu.ru; 2 Komarov Botanical Institute of Russian Academy of Sciences, St. Petersburg 197022, Russia; npetrova@binran.ru; 3 Institute of Chemical Biology and Fundamental Medicine, SB RAS, Novosibirsk 630090, Russia; alexander.chernonosov@niboch.nsc.ru (A.A.C.); koval@niboch.nsc.ru (V.V.K.); 4 Department of Natural Sciences, Novosibirsk State University, Novosibirsk 630090, Russia; 5 State Key Laboratory of Systematic and Evolutionary Botany, Institute of Botany, Chinese Academy of Sciences, Beijing 100093, China; wangwei1127@ibcas.ac.cn; 6 College of Life Sciences, University of Chinese Academy of Sciences, Beijing 100049, China

**Keywords:** *Anemonopsis macrophylla*, liquid chromatography–high-resolution mass spectrometry (LC-HRMS), metabolome, flavonoid, coumarin, furochromone, phenolcarboxylic acid

## Abstract

*Anemonopsis* Siebold et Zucc. is an unstudied single-species genus belonging to the tribe Cimicifugeae (Ranunculaceae). The only species of this genus—*Anemonopsis macrophylla* Siebold and Zucc.—is endemic to Japan. There are no data on its chemical composition. This work is the first to determine (with liquid chromatography–high-resolution mass spectrometry, LC-HRMS) the chemical composition of methanol extracts of leaves and flowers of *A. macrophylla*. More than 100 compounds were identified. In this plant, the classes of substances are coumarins (13 compounds), furocoumarins (3), furochromones (2), phenolic acids (21), flavonoids (27), and fatty acids and their derivatives (15 compounds). Isoferulic acid (detected in extracts from this plant) brings this species closer to plants of the genus *Cimicifuga*, one of the few genera containing this acid and ferulic acid at the same time. Isoferulic acid is regarded as a reference component of a quality indicator of *Cimicifuga* raw materials. The determined profiles of substances are identical between the leaf and flower methanol extracts. Differences in levels of some identified substances were revealed between the leaf and flower extracts of *A. macrophylla*; these differences may have a substantial impact on the manifestation of the biological and pharmacological effects of the extracts in question.

## 1. Introduction

The tribe Cimicifugeae Torrey and Gray is included in the family Ranunculaceae Juss., represented by four genera and more than 49 plant species [1,2,3,4]. These genera include *Anemonopsis* Siebold et Zucc. (one species), *Actaea* L. (32 species), *Eranthis* Salisb. (14 species), and *Beesia* Balf. f. et W. W. Sm. (two species), which have a circumboreal distribution. The larger genera occupy wide ranges in temperate forests or plains throughout Eurasia (*Eranthis*) or Eurasia and North America (*Actaea*). Both *Anemonopsis macrophylla* Siebold and Zucc., and the two species of *Beesia* occupy restricted forest habitats: *Anemonopsis* represents an island community of Honshu, Japan, and the two *Beesia* species inhabit the Sino-Himalayan mountains of East Asia [5].

**Figure 1:**
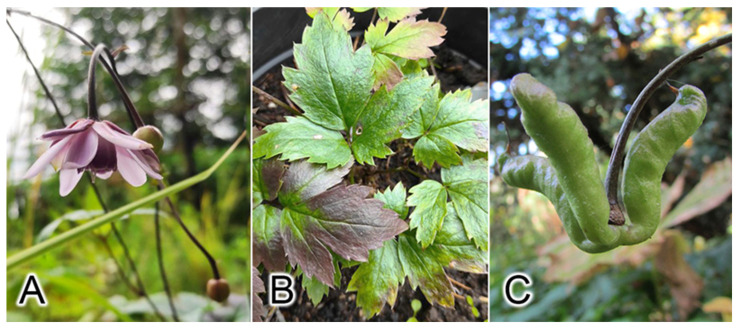
*A. macrophylla*: (**A**) flowers, (**B**) leaves, and (**C**) a fruit. Photo by Andrey S. Erst.

*Anemonopsis* is a monotypic and endemic genus of Japan and grows in temperate deciduous forests in central Honshu [6]. *A. macrophylla* is a perennial plant characterized by 2–4-ternate basal and lower cauline leaves, irregularly incised-dentate leaflets, a loosely racemiform 3–8-flowered definite inflorescence, nutant flowers with slender pedicels and with 7–10 reddish purple sepals; 10 or more erect, concolorous with sepals, without a nectary, petals anchor-shaped with 2–4 follicles (fruits), bending down fruiting pedicels, follicles rising up with elongate stalks and squamate seeds (Figure 1). This genus and *Actaea* differ from *Eranthis* and *Beesia* by compound, not undivided simple leaves but are similar to *Eranthis* in terms of a nonrecemiform or laxly recemiform inflorescence (with at most several flowers or a single flower) or a nondensely racemiform inflorescence (with many flowers) as in *Actaea* [7]. According to morphological and molecular phylogenetic data, *Anemonopsis* and *Beesia* form a separate clade that is sister to the *Eranthis* + *Actaea* clade. [8]. A morphological analysis of Loconte et al. [9] was influenced by a reduction in leaf complexity in *Beesia* and *Eranthis* as compared with *Actaea* and *Anemonopsis* [1]. Nonetheless, leaf, floral, and fruiting characteristics in both *Beesia* and *Eranthis* provide a new basis for tribal redefinition inferred from a unique combination of traits, which are as follows: a rhizomatous or tuberous type of plant; actinomorphic flowers that are stand-alone or borne by racemes or by racemose panicles; free, follicular, or baccate fruits with many seeds; one to several follicles with transverse external venation; and simple or ternately compound leaves [5].

In addition to morphological characteristics, chemosystematic traits could clarify the position of *A. macrophylla* in the tribe Cimicifugeae; however, research on the chemical composition of *A. macrophylla* has not been conducted to date. Studying the chemical composition of *A. macrophylla* is of interest not only because the obtained information (along with morphological, anatomical, and other characteristics) can be used in plant taxonomy but also due to practical interest: this knowledge makes it possible to search for plants that are promising producers of biologically active compounds. LC-HRMS (liquid chromatography–high-resolution mass spectrometry) is one of the most effective and fastest methods for analyzing metabolites in a multicomponent mixture (such as plant extracts) [10,11]. 

The aim of this study was to analyze methanol extracts from leaves and flowers of *A. macrophylla* for metabolites by LC-HRMS.

## 2. Results and Discussion

### 2.1. Analysis of the Set of Bioactive Compounds in the A. macrophylla Extracts by LC-HRMS

Methanol extracts from leaves and flowers of *A. macrophylla* were found to contain a large number of biologically active components (Table 1). Using LC-HRMS and several databases, it was possible to identify more than 100 compounds in extracts from these organs of the species under study. Among them, we identified 27 flavonoids, 21 phenolic acids, 16 coumarins, 15 fatty acids and derivatives, 10 organic acids, eight amino acids, five triterpenoids, two furochromones, and one sugar. The profiles of identified substances do not differ between the leaves and flowers of this plant.

#### 2.1.1. Coumarins

At least 16 coumarins were found in the extracts from *A. macrophylla*. They are mainly represented by simple coumarins, and only three of them are furocoumarins.

Coumarins are unsaturated aromatic lactones based on a benzene cycle fused to an α-pyrone ring [12]. The simplest compound is coumarin (**7**) (2H-1-benzopyran-2-one), first isolated in 1820 independently by A. Vogel and by N. Guibourt from *Coumarouna odorata* Aubl. [*Dipteryx odorata* (Aubl.) Willd.].

**Figure 2:**
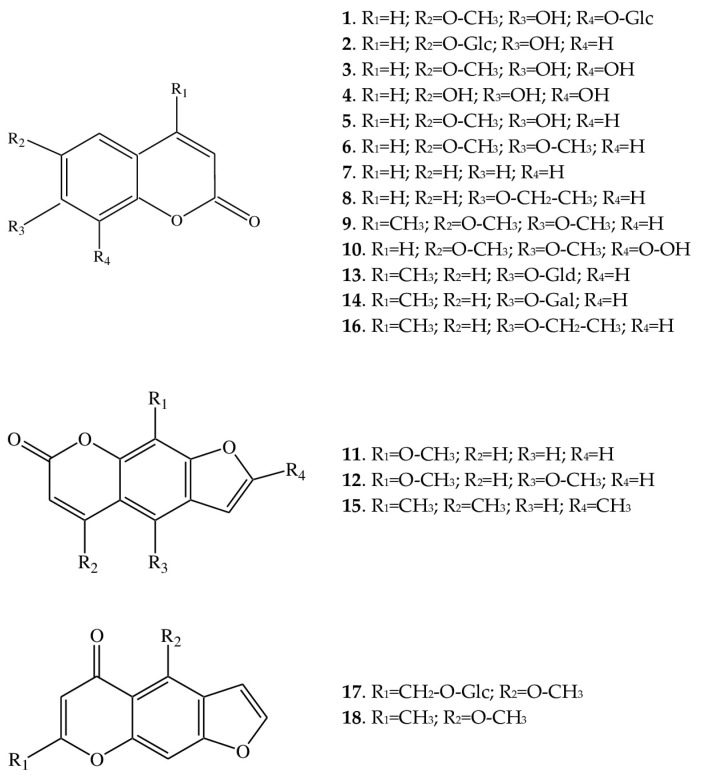
Structures of coumarins (**1**–**10**, **13**, **14**, and **16**), furocoumarins (**11**, **12**, and **15**), and furochromones (**17** and **18**) from *A. macrophylla*.

Coumarin is well known not only for serving as the basis for the synthesis of a whole class of compounds but also for the pleasant smell of freshly cut hay [13]. It is believed that coumarin is absent in undamaged plant tissues but is formed from melilotoside when cells are damaged [14]. In contrast to coumarin itself (**7**), which has no substituents, other coumarins identified in *A. macrophylla* have substituents at the C-6, C-7, or C-8 positions (Figure 2). 

Such substituents are one or several hydroxyl groups [in fraxetin (**3**), fraxin (**1**), esculin (**2**), scopoletin (**5**), and esculetin (**4**)], an ethoxy group [in 7-ethoxycoumarin (**8**) and maraniol (**16**)], or a methyl- and/or methoxy group [in fraxetin (**3**), fraxin (**1**), scoparone (**6**), maraniol (**16**), and 6,7-dimethoxy-4-methylcoumarin (**9**)]. 8-Acetyl-6,7-dimethoxycoumarin (**10**), besides two methoxy groups, contains an acetyl group. In addition, two coumarins have glucuronic acid [4-methylumbelliferyl-β-glucuronide (**13**)] or galactopyranose [4-methylumbelliferyl-β-galactopyranoside (**14**)] as substituents at the C-7 position. Thus, the diversity of coumarin compounds in *A. macrophylla* is achieved via the addition of the above-mentioned functional groups. Simple coumarins are coumarins in which hydrogens at the C-6, C-7, or C-8 position only on the benzene ring are replaced by hydroxyl, methoxyl, isopentenyl, or some other groups without the formation of furan or pyran rings.

Furocoumarins are formed by the addition of furan to a simple coumarin either at the C-6 and C-7 or at the C-7 and C-8 positions, whereas pyranocoumarins are formed via the attachment of pyran. We did not find pyranocoumarins in *A. macrophylla*, but from the subclass of furocoumarins, we were able to identify methoxsalen (**11**), trioxsalen (**15**), and isopimpinellin (**12**). All three compounds are based on 7-H-furo [3,2-g]chromen-7-one; although methoxsalen carries an additional methoxy group at the C-8 position, isopimpinellin has two methoxy groups (at C-5 and C-8 positions), whereas trioxsalen contains methyl groups at positions C-4, C-8, and C-5’. Furocoumarins can be linear or angular compounds [15]; only linear furocoumarins were present in the analyzed extracts.

Furthermore, we detected 4-hydroxycoumarin in the extracts of *A. macrophylla*. This compound contains a hydroxyl group as a substituent, but it is located at the C-4 position, which is rare because substituents are most often present at positions C-6, C-7, and/or C-8. Additionally, in the literature, it was found that 4-hydroxycoumarin derives not from coumarin but directly from melilotoside as a result of metabolic processes involving *Penicillium* and some *Aspergillus* species [16]. For this reason, we did not include this compound in the list of substances (Table 1) identified in the extracts of *A. macrophylla*.

The coumarins found in *A. macrophylla* are quite widespread in the plant kingdom, and, in general, this class of secondary metabolites has now been detected in almost 30 families and more than 150 species. Representatives of such families as Rutaceae Juss., Clusiaceae Lindl., Apiaceae Lindl., and some others are rich in coumarins [17]. Coumarins have also been registered as representatives of the family *Ranunculaceae* Juss. [18]. As for the occurrence of coumarins in the genera of the tribe Cimicifugeae, which includes *Anemonopsis*, it is difficult to say anything because these plants have rarely been investigated by phytochemists. Continued research involving more genera closely related to *Anemonopsis* will allow for a more definitive assessment of the taxonomic significance of these compounds. Nonetheless, coumarins have been found in some species of *Actaea* L., *Cimicifuga* Wernisch., and *Eranthis* Salisb. [4,10,19,20]. The search for new plants containing coumarins is important because, in many natural coumarins, including those found in *A. macrophylla* (scopoletin and fraxetin), an anti-inflammatory activity has been detected [21,22,23,24]. A tight structure–activity relationship has also been documented for coumarins, thus making it possible to use coumarin molecules as the basis for the development of various pharmaceuticals [25,26].

#### 2.1.2. Furochromones

From the furochromones class, khelloside (**17**) and visnagin (**18**) were identified in the extract. Both substances are based on furochromone structure with a methoxy group at position C-4, but in visnagin, a methyl group is located at position C-7, whereas in khelloside, there is a glucopyranoside at this position. In the last decade, this subclass of compounds has attracted research interest: e.g., reviews have been published on the natural diversity of furochromones [27], their biological activity [28], and their use in medicinal chemistry as fluorescent probes [29].

#### 2.1.3. Phenolic Acids

**Figure 3:**
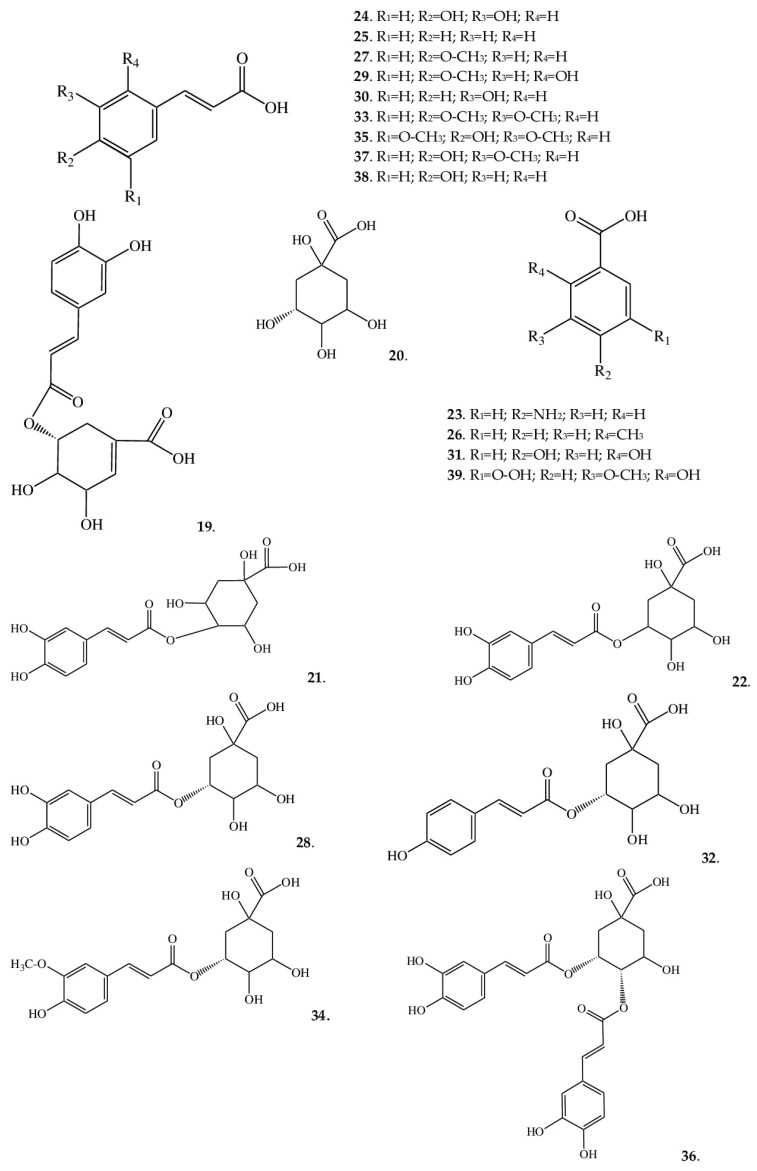
Structures of phenolic acids from *A. macrophylla*.

In the extracts of *A. macrophylla*, the hydroxycinnamic-acid subclass proved to be highly diverse (Figure 3). Cinnamic acid (**25**) is an aromatic carboxylic acid and is a key compound present in many medicinal plants [30]. In nature, it occurs in *cis*- and *trans*-forms, the latter being the most common. Cinnamic acid has a variety of biological properties, allowing the creation of effective CNS-stimulatory, immunostimulatory, and antimicrobial drugs on its basis. There is evidence that the cinnamoyl moiety is crucial for the manifestation of antiradical activity, whereas the nature and position of substituents on the aromatic ring give only an increase or decrease in activity [31]. Nevertheless, it is more often reported that the introduction of additional hydroxyl substituents into this acid enhances the reducing properties; this is because the mobile proton of the OH group is the primary center of inhibition of a radical [32,33]. Even the presence of a methoxy group can enhance the antiradical properties [34,35].

In the *A. macrophylla* extracts, the diversity of cinnamic acid derivatives has several reasons: additional substituents in the form of hydroxyls [at the C-4 position in *p*-coumaric acid (**38**) or at positions C-3 and C-4 as in caffeic acid (**24**)] or methoxy groups [at position C-3 and position C-4 in 3,4-dimethoxycinnamic acid (**33**)]. Both hydroxy and methoxy groups can be attached simultaneously [as in 2-hydroxy-4-methoxycinnamic acid (**29**), sinapinic acid (**35**), ferulic acid (**37**), and isoferulic acid (**27**)]. Furthermore, when combined with quinic acid, cinnamic acid gives rise to a number of acids: neochlorogenic (**22**), cryptochlorogenic (**21**), and accordingly chlorogenic (**28**) acids. Chlorogenic acid is the most common in nature, but the combination of two different acids in its chemical structure [ester of caffeic acid and (–)-quinic acid] explains the high antioxidant activity [36]. Further, when combined with quinic acid, cinnamic acid gives such derivatives as 3-*O*-feruloylquinic (**34**), 5-coumaroylquinic (**32**), and 4,5-dicaffeoylquinic acids (**36**).

Aside from the fact that cinnamic acid itself has various types of activity, it has many derivatives: no less valuable substances that also have a wide range of pharmacological activities, such as antibacterial [37], antifungal [38], neuroprotective [39], and anticancer effects [40]. For this reason, they have been separated into an independent class of biologically active substances and have affected the chemical classification of medicinal plants. Furthermore, derivatives of cinnamic acid are intermediates in the synthesis of such compounds as stilbenes and styrenes and participate in the biosynthesis of lignin and other compounds. The structural features of derivatives of cinnamic acid (unsaturation and several hydroxylic and/or carboxylic groups) have even enabled their use in the synthesis of polymers: polyesters, polyamides, and polyanhydride esters, which in turn have found applications in industrial engineering and medical fields [41].

In *A. macrophylla*, aside from derivatives of cinnamic acid, we detected acids based on benzoic acid. The latter is a monobasic aromatic carboxylic acid that possesses high reactivity, resulting in a wide variety of compounds [30]. Depending on the characteristics of chemical structure, such substances have found applications in the food, perfume, and pharmaceutical industries. Numerous pharmacological studies have shown that benzoic acid derivatives that contain, for example, a carboxyl group is promising as potential diuretics, and carbamide derivatives hold promise as analgesics [42]. In the extract analyzed here, benzoic acid derivatives are represented by 2-methylbenzoic acid (**26**), 2,4-dihydroxybenzoic acid (**31**), 4-aminobenzoic acid (**23**), and 5-carboxyvanillic acid (**39**).

Most of the acids, among those detected in the extracts of *A. macrophylla* are widespread among plants, but, for example, isoferulic acid is much less common. For instance, the genus Cimicifuga (closely related to *Anemonopsis*) is one of the few genera containing both ferulic and isoferulic acids. At least three species of this genus (*C. dahurica*, *C. foetida*, and *C. heracleifolia*) are listed in the Pharmacopoeia of the People’s Republic of China, and their pharmacological properties are attributed to the presence of isoferulic acid in extracts. Additionally, this compound is regarded as a reference component of a quality indicator of Cimicifuga raw materials [43]. The actions of isoferulic acid as an anti-inflammatory and antiviral ingredient have been confirmed [44,45], but its antidiabetic effects are the most interesting [46]. Further, in Cimicifuga species, a number of phenolic acids have been registered: cimicifugic acids A–N, cimiracemates A–D, and others, which are exclusive chemical constituents of the genus Cimicifuga [47]. These phenolic compounds are formed via the condensation of piscidic acid or fukiic acid with isoferulic, ferulic, caffeic, 3,4-dihydroxybenzoic, or some other acids [48]. Further research is needed to verify whether *A. macrophylla* synthesizes such acids.

#### 2.1.4. Flavonoids

**Figure 4:**
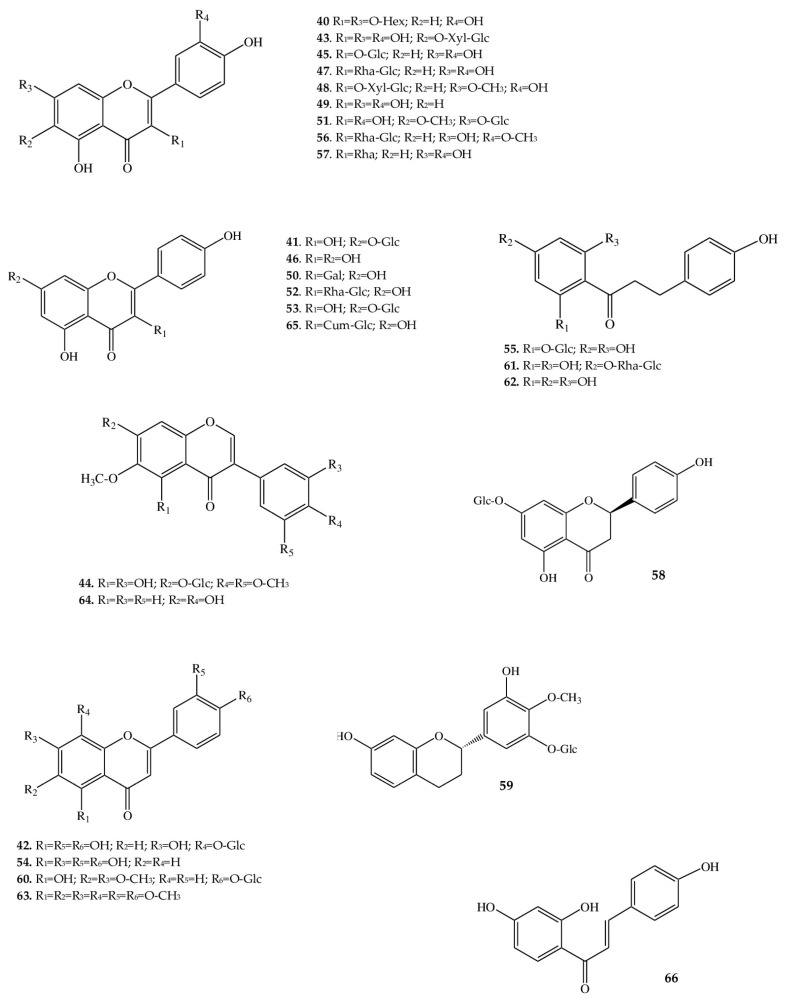
Structures of flavonoids from *A. macrophylla*.

One of the major classes of secondary metabolites produced by *A. macrophylla* is flavonoids (Table 1). We revealed and identified 27 compounds belonging to this class, with flavonols being the most diverse (Figure 4). The flavonols found in *A. macrophylla* are based on quercetin (**40**), kaempferol (**46**), and their derivatives. The following quercetin glycosides were identified in the assayed samples: quercetin-3β-glucoside (**45**), quercetin-6-*O*-β-xylopyranosyl-β-glucopyranoside (**43**), rutin (**47**), patulitrin (**51**), narcissin (**56**), rhamnetin-3-*O*-xylopyranosyl-glucopyranoside (**48**), and dihydroquercetin-3-rhamnoside, better known as astilbin (**57**). Additionally, a flavonoid (**40**) was found whose aglycone is quercetin that has substituents at positions C-3 and C-7 in the form of hexose sugars, which we were unable to identify. Among derivatives of kaempferol, the following glycosides were noted: trifolin (**50**), kaempferol-7-*O*-glucoside (**53**), dihydrokaempferol-7-*O*-glucoside (**41**), nicotiflorin (**52**), and tiliroside (**65**).

Analysis of our data allowed us to identify isoflavones and flavans in the extract, for which the following structural interpretation was proposed: iridin (**44**), glycitein (**64**), and auriculoside (**59**). Flavanones are represented by prunin (**58**), flavones by luteolin (**54**) and nobiletin (**63**), and glucosides by cirsimarin (**60**) and gossypin (**42**), which contain glucopyranose as a sugar residue.

Dihydrochalcones are also phenolic compounds with a flavonoid backbone but are characterized by the absence of heterocyclic ring C. The small set of dihydrochalcones here consists of four compounds: naringin dihydrochalcone (**61**), phloretin (**62**), and its glycosides phlorizin (**55**) and isoliquiritigenin (**66**). The latter compound is most often registered in underground parts of members of the family Fabaceae [49], but there is evidence of its identification in Ranunculaceae, too [50]. Isoliquiritigenin possesses a chalcone skeleton with three hydroxyl groups (at positions C-4, C-2’, and C-4’): such a hydrophobic compound is almost insoluble in water. Isoliquiritigenin is of particular interest not only as a compound from the class of chalcones (precursors of many flavanones [51]) but also as a substance that manifests antidiabetic [52], spasmogenic [53], anticancer [54], vasorelaxant [55], and other types of activity. Phloretin (**62**), just like isoliquiritigenin, contains two aromatic phenolic rings and a carbonyl group, but in addition to the three hydroxyl groups, there is another one at the C-6’ position. Phloretin has a wide range of pharmacological effects on the human body and is devoid of toxicity [56].

#### 2.1.5. Other Classes of Compounds

In addition, amino acids (**87**–**94**), sugars (**95**), and organic acids (**96**–**105**) were detected in the extracts of *A. macrophylla*. Five compounds were identified as triterpenoids and matched ursolic acid (**67**), lupenone (**68**), oleanolic acid (**69**), cucurbitacin I (**70**), and cucurbitacin S (**71**). Several free saturated and unsaturated fatty acids and their derivatives were found, with chain lengths ranging from six carbon atoms (adipic acid) to 22 carbon atoms (docosahexaenoic acid) (**72**–**86**). The most numerous here are fatty acids with a chain length of 18 carbons.

### 2.2. A Comparative Analysis of Concentrations of the Identified Compounds between Leaf and Flower Methanol Extracts from A. macrophylla

**Figure 5:**
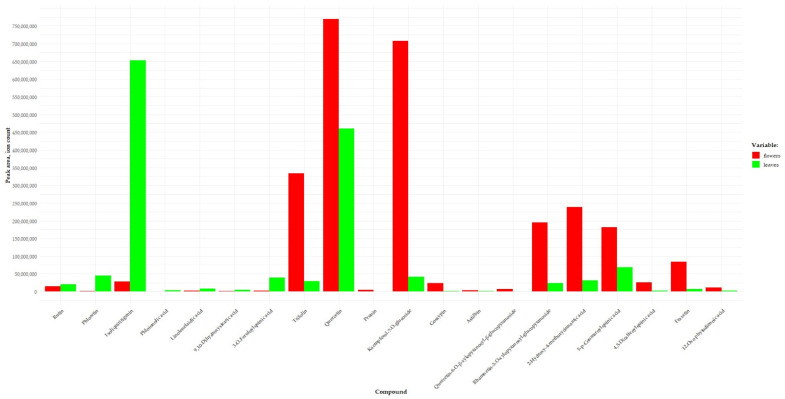
Peak area of substances identified in *A. macrophylla* leaves and flowers.

Plant extracts having identical metabolite profiles may possess different pharmacological and biological activities. The manifestation of one or another activity is influenced not only by the profile of biologically active substances but also by their levels [57,58]. It was determined that the profiles of the identified substances are identical between the leaves and flowers of *A. macrophylla*. The comparative analysis of peak areas revealed differences in relative concentrations of some substances between the leaves and flowers of this species at a significance level of *p* ≤ 0.05. Levels of the following flavonoids turned out to be higher in the leaves: flavonol rutin (substance peak area ratio leaves/flowers = 1.46), chalcones [phloretin (ratio = 52.30) and isoliquiritigenin (23.32)], fatty acids [phloionolic acid (7.19), linolenelaidic acid (3.23), and 9,10-dihydroxystearic acid (2.92)], and a phenolcarboxylic acid [3-*O*-feruloylquinic acid (13.89)] as compared to concentration of these compounds in methanolic extracts from the plant’s flowers (Figure 5). Flowers of *A. macrophylla* are distinguished by higher concentrations of the following flavonols: quercetin (substance peak area ratio flowers/leaves = 1.67) and its derivatives [quercetin-6-*O*-β-xylopyranosyl-β-glucopyranoside (ratio = 30), rhamnetin-3-*O*-xylopyranosyl-glucopyranoside (8.30), and astilbin (2.85)], kaempferol derivatives [trifolin (11.24) and kaempferol-7-*O*-glucoside (16.86)], a flavanone [prunin (8.13)], a flavone [gossypin (17.76)], phenylcarboxylic acids [2-hydroxy-4-methoxycinnamic acid (7.68), 5-p-coumaroylquinic acid (2.69), and 4,5-dicaffeoylquinic acid (9.40)], a coumarin [fraxetin (13.24)], and a fatty acid [12-oxo-phytodienoic acid (4.45)] (Figure 5). The observed differences in concentrations of substances between the leaves and flowers may considerably affect the biological and pharmacological activities of the extracts.

## 3. Materials and Methods

### 3.1. Plant Material and Preparation of the Extract

Live material of *A. macrophylla* was collected in Japan: Saitama Prefecture, Chichibu Shi City, Shiroku, near a village, 340 m above sea level, 35.956556° N, 138.987667° E, by A.S. Erst, T.V. Erst, and H. Ikeda, 2 April 2019 (NS, s.n.). Leaves and flowers were collected from plants cultivated in the Central Siberian botanical garden RAS in 2023. Original live materials were collected from the natural population of Japan in 2019. The plant under study was collected and identified by an expert on the Ranunculaceae family: A.S. Erst, Ph.D. (a senior researcher at the Central Siberian Botanical Garden SB RAS) using the phenotype and morphological traits (voucher specimens No. AM-J-109). The material was collected from 5–10 typical specimens of *A. macrophylla*. The collected material was dried in silica gel.

Air-dried plant material was mechanically ground up to obtain a homogeneous powder with particle size as small as 0.5 mm. The methanol extract was prepared as follows: 40 mg of crushed leaves or flowers were placed in a 2 mL Eppendorf tube, then 800 μL of 96% methanol was added and mixed for 30 s on an IKA vortex 3 (IKA-Werke GmbH and Co. KG, Staufen, Germany). Next, the extracts were sonicated for 30 min in a UM-2 ultrasonic bath (Unitra-Unima Olsztyn, Poland) and then agitated for 30 min on a TS-100C thermal shaker (Biosan, Riga, Latvia). The resulting eluates were centrifuged for 1 min on a MiniSpin^®^ plus centrifuge (Eppendorf, Hamburg, Germany) at 14,000× *g*. An aliquot of the supernatant was transferred to a clean, dry 2 mL Eppendorf tube. The remaining material was again covered with 800 μL of 96% methanol, and the procedure was repeated. Before the assay, the combined extract was centrifuged in an Eppendorf 5425 centrifuge (Eppendorf, Hamburg, Germany) at 15,000× *g* and passed through a membrane filter with a pore diameter of 0.45 μm.

### 3.2. LC-HRMS Analysis of Metabolites in the A. macrophylla Extracts

LC-HRMS was conducted at the Core Facility of Mass Spectrometric Analysis at the Institute of Chemical Biology and Fundamental Medicine SB RAS (Novosibirsk, Russia).

An Ultimate 3000 liquid chromatograph (Thermo Fisher Scientific, San Jose, CA, USA) coupled with a Q Exactive HF mass spectrometer (Thermo Fisher Scientific, San Jose, CA, USA) was utilized to determine metabolomic profiles of the *A. macrophylla* extracts. The chromatographic separation was carried out at a 0.4 mL/min flow rate on a Zorbax Eclipse XDB-C8 reversed-phase column (150 mm × 3.0 mm, 5 μm, Agilent Technologies, Santa Clara, CA, USA) thermostatted at 40 °C. The mobile phase was composed of 0.1% aqueous formic acid (eluent A) and 0.1% formic acid in acetonitrile (eluent B). The elution gradient was implemented as follows: from minute 0 to minute 1, 5% B; then 40 min from 5% to 70% B; followed by an increase to 100% B for 10 min; 100% B for 8 min; a decrease to 5% B for 2 min; and re-equilibration under the initial conditions for 10 min.

The settings of the electrospray ionization (ESI) source were as follows: electrospray voltage: 3.2 kV in the negative mode and 4.2 kV in the positive mode; capillary temperature: 350 °C; and the S lens RF level: 50. Data were obtained using two methods: the full-scan was used for compound detection, and full-scan data-dependent acquisition (FS-dd-MS2) was applied for the compounds’ identification. The scan was performed in positive and negative modes at a resolving power of 120,000 full-width at half maximum (FWHM) for *m*/*z* 200. The following settings of the mass spectrometer were employed: scan range: *m*/*z* 67–900; automatic gain control (AGC): 1e6; and injection time: 100 ms. A targeted tandem mass spectrometry (MS/MS, i.e., dd-MS2) analysis was performed in both positive and negative modes at 15,000 FWHM (*m*/*z* 200), and the isolation window was *m*/*z* 2.0. Normalized collision energy for the fragmentation of molecular ions was set to 20, 50, and 100 eV. AGC for dd-MS2 was set to 1 × 10^5^, with an injection time of 50 ms and a loop count of 5. In the dd settings section, the AGC target was programmed at 5 × 10^3^, and the maximum injection time was set to 50 ms. The data were analyzed using Xcalibur 4.0 and Compound Discoverer 3.1 software (Thermo Fisher Scientific, San Jose, CA, USA). All the samples, including blank samples, were assayed in triplicate. All the samples were processed in Compound Discoverer 3.1 via a common workflow called “Environmental Unknown ID w Online and Local Database Searches”. A mass tolerance of 10 ppm was applied to all nodes. Several databases, i.e., KEGG (https://www.genome.jp/kegg/; last accessed 5 November 2023), MassBank (https://massbank.eu/MassBank/; last accessed 5 November 2023), PlantCyc (https://plantcyc.org/; last accessed 5 November 2023), Planta Piloto de Quimica Fina Universidad de Alcala (http://www.cqab.eu/index.php/en/; last accessed 5 November 2023), AraCyc (https://www.arabidopsis.org/biocyc/; last accessed 5 November 2023), Extrasynthese (https://www.extrasynthese.com/; last accessed 5 November 2023), Golm Metabolome Database (last accessed 5 November 2023), Indofine (https://www.indofinechemical.com/; last accessed 5 November 2023), and Sequoia Research Products (http://www.chemcd.com/supplier/sequoia.html; last accessed 5 November 2023) were chosen in ChemSpider. A more detailed procedure for identifying substances is described in Ref. [10].

Several mzVaults were used in the mzVault Search node: Creation of a Plant Metabolite Spectral Library for Untargeted and targeted Metabolomics.db [59], Negative ion mode_Jan2021.db, Positive ion mode_Jan2021.db (https://more.bham.ac.uk/bamcg/resources/; last accessed 5 November 2023), and MS2_library.db (https://doi.org/10.1016/j.jchromb.2020.122105, last accessed 27 November 2023).

Metabolites were identified on the basis of both accurate mass and fragment mass “fingerprint” spectra via searches against the spectra of compounds available in the mzCloud database (https://www.mzcloud.org; last accessed 5 November 2023). If compounds were absent in mzCloud, they were tentatively identified using a ChemSpider search. According to the workflow, the masses extracted from the chromatograms were aligned and filtered to remove (i) background compounds present in the blank sample and (ii) compounds’ masses that were absent in the databases.

### 3.3. Statistical Analysis

This analysis was carried out in R. All samples, including blank samples, which consisted of the pure solvent, were analyzed as two biological replicates with three technical replicates per treatment group.

## 4. Conclusions

This study shows that the only representative of the genus *Anemonopsis* (*A. macrophylla*) synthesizes and accumulates a number of secondary metabolites of a phenolic and nonphenolic nature. Metabolites of this endemic plant of Japan were analyzed for the first time using the LC-HRMS method. In methanol extracts from flowers and leaves of *A. macrophylla*, 105 metabolites of various chemical classes were identified. Structural diversity was noted for phenolic acids and flavonoids. Further, simple coumarins and furocoumarins, as well as furochromones, were found in the chemical profile of *A. macrophylla*. The data presented in this work indicate that *A. macrophylla* is a rich source of secondary metabolites and that this plant can be used for research on (and production of) new biologically active fractions and individual compounds. Furthermore, it was established that the phytochemical uniqueness of individual organs is primarily determined using the quantitative characteristics of metabolites, not by their qualitative profile. Differences in concentrations of some identified substances between the extract from leaves and the extract from flowers of *A. macrophylla* may have a substantial impact on the manifestation of the biological activity of the extracts in question.

## Figures and Tables

**Table 1:** Chemical constituents tentatively identified in methanol extracts from *A. macrophylla* leaves and flowers by LC-HRMS using databases mzCloud, mzVault, and ChemSpider.

ID	Identified Compounds	t_R_ (min)	Calcd. Mass (Da)	Found Mass (Da)	Delta of Mass (ppm)	Fragmentation (*m*/*z*)	Score	Mode	L	Fl
Coumarins										
1	Fraxin	5.60	370.090	370.089	−2.11	207.029, 163.039	61.9 ^1^	Negative	+	+
2	Esculin	6.20	340.079	340.079	−1.65	177.018	78.9 ^1^/95.8 ^2^	Negative	+	+
3	Fraxetin	6.38	208.037	208.034	−1.29	163.039, 149.096, 135.044	-	Positive	+	+
4	Esculetin	10.57	178.027	178.027	1.91	-	-	Positive	+	+
5	Scopoletin	11.98	192.042	192.042	0.77	-	-	Positive	+	+
6	Scoparone	13.39	206.058	206.058	1.79	-	-	Positive	+	+
7	Coumarin	21.45	146.037	146.037	1.37	119.050, 105.070, 91.055, 66.039	77.0 ^2^	Positive	+	+
8	7-Ethoxycoumarin	23.90	190.063	190.063	0.90	121.103, 93.072	61.7 ^2^	Positive	+	+
9	6,7-Dimethoxy-4-methylcoumarin	23.91	220.074	220.074	1.04	-	-	Positive	+	+
10	8-Acetyl-6,7-dimethoxycoumarin	25.05	248.068	248.069	0.42	-	-	Positive	+	+
11	Methoxsalen	26.92	216.042	216.043	1.81	-	-	Positive	+	+
12	Isopimpinellin	27.15	246.053	246.053	0.51	-	-	Positive	+	+
13	4-Methylumbelliferyl- glucuronide	27.20	352.079	352.080	1.71	-	-	Positive	+	+
14	4-Methylumbelliferyl- galactopyranoside	28.52	338.100	338.100	0.30	-	-	Positive	+	+
15	Trioxsalen	33.39	228.079	228.079	0.07	-	-	Positive	+	+
16	Maraniol	40.83	204.079	204.079	1.18	-	-	Positive	+	+
Furochromones										
17	Khelloside	8.33	408.106	408.103	−5.70	-	-	Positive	+	+
18	Visnagin	24.05	230.058	230.058	0.60	-	-	Positive	+	+
Phenolic acids										
19	5-Caffeoylshikimic acid	1.78	336.085	336.084	−1.64	178,034 161,023 135,044	-	Negative	+	+
20	Quinic acid	1.84	192.063	192.062	−4.57	111.008, 93.033, 87.008, 85.026	87.7 ^1^/90.2 ^2^	Negative	+	+
21	Cryptochlorogenic acid	1.84	354.095	354.095	−0.92	163.039, 145.026, 135.044	86.8 ^1^	Positive	+	+
22	Neochlorogenic acid	2.40	354.095	354.095	−1.48	191.055, 179.034, 173.045, 135.044	88.4 ^1^/94.7 ^2^	Negative	+	+
23	4-Aminobenzoic acid	5.51	137.048	137.048	−0.39	111.044, 93.034, 65.039	74.6 ^2^	Positive	+	+
24	Caffeic acid	5.78	180.042	180.042	0.28	163.039, 145.028, 135.044, 117.035, 107.049	89.7 ^1^/91.2 ^2^	Positive	+	+
25	Cinnamic acid	6.64	148.052	148.053	1.05	131.050, 103,055	-	Positive	+	+
26	2-Methylbenzoic acid	7.12	136.052	136.051	−8.48	135.044, 117.034, 107.049	80.3 ^1^	Negative	+	+
27	Isoferulic acid	7.49	194.058	194.058	1.42	177.055, 163.039, 145.028, 117.034	83.4 ^1^	Positive	+	+
28	Chlorogenic acid	7.71	354.095	354.095	−0.55	183.039, 145.029, 136.044	89.7 ^1^/92.5 ^2^	Positive	+	+
29	2-Hydroxy-4-methoxycinnamic acid	8.90	194.058	194.058	0.75	177.054, 153.054, 133.064	75.6 ^1^	Positive	+	+
30	2-Hydroxycinnamic acid	9.30	164.047	164.046	−6.37	119.049	82.6 ^1^	Negative	+	+
31	2,4-Dihydroxybenzoic acid	9.71	154.027	154.026	−6.83	109.028, 66.038, 67.018	77.2 ^1^/92.4 ^2^	Negative	+	+
32	5-*p*-Coumaroylquinic acid	9.76	338.100	338.100	−0.59	191.055, 173.044, 163.039, 93.033	83.0 ^1^	Positive	+	+
33	3,4-Dimethoxycinnamic acid	9.96	208.074	208.074	0.44	191.107, 163.112	53.6 ^2^	Negative	+	+
34	3-*O*-Feruloylquinic acid	10.74	368.111	368.111	−0.30	191.055, 134.037, 93.033	85.4 ^1^	Positive	+	+
35	Sinapinic acid	12.67	224.068	224.068	−3.48	208.037, 193.013, 164.049	84.2 ^1^	Negative	+	+
36	4,5-Dicaffeoylquinic acid	15.18	516.127	516.126	−0.58	191.056, 179.034, 173.045, 136.044	77.2 ^1^	Negative	+	+
37	Ferulic acid	15.61	194.058	194.057	−4.64	193.050, 178.027, 134.038	83.2 ^1^	Negative	+	+
38	(E)-*p*-Coumaric acid	21.45	164.047	164.048	1.60	147.044, 119.049	-	Positive	+	+
39	5-Carboxyvanillic acid	24.03	212.032	212.032	0.89	-	-	Positive	+	+
Flavonoids										
40	Quercetin 3,7-dihexoside	6.67	626.148	626.148	−0.86	-	-	Positive	+	+
41	Dihydrokaempferol-7- glucoside	8.67	450.116	450.116	−0.67	287.056, 259.060, 178.998, 125.023	84.1 ^1^	Negative	+	+
42	Gossypin	10.96	480.090	480.090	−1.07	-	-	Positive	+	+
43	Quercetin-6-*O*- xylopyranosyl- glucopyranoside	11.17	596.138	596.138	−0.04	300.027, 271.025, 255.029, 178.998, 151.003	87.8 ^1^	Negative	+	+
44	Iridin	11.28	522.137	522.137	−1.31	-	-	Negative	+	+
45	Quercetin-3-glucoside	11.62	464.095	464.095	−0.03	303.050, 285.040, 229.047, 137.023, 85.029	93.1 ^1^	Positive	+	+
46	Kaempferol	11.75	286.048	286.048	0.03	287.549, 241.019, 213.055, 165.018, 153.018	97.1 ^1^	Positive	+	+
47	Rutin	11.99	610.153	610.153	0.11	300.027, 271.025, 255.029, 178.998, 151.003	92.6 ^1^	Negative	+	+
48	Rhamnetin-3-*O*-xylopyranosyl-glucopyranoside	12.21	610.153	610.153	−0.34	314.043, 299.019, 271.025, 243.030	83.5 ^1^	Negative	+	+
49	Quercetin	12.43	302.043	302.043	0.08	303.050, 285.039, 257.045, 229.050, 165.018, 137.023	98.6 ^1^	Positive	+	+
50	Trifolin	12.97	448.101	448.100	−0.79	304.054, 287.055, 85.029, 61.028	87.5 ^1^	Positive	+	+
51	Patulitrin	13.34	494.106	494.105	−1.38	-	-	Negative	+	+
52	Nicotiflorin	13.51	594.158	594.158	−0.81	287.055, 85.029	-	Positive	+	+
53	Kaempferol-7-*O*- glucoside	13.54	448.101	448.100	−0.79	304.054, 287.055, 119.086	82.1 ^1^	Positive	+	+
54	Luteolin	13.55	286.048	286.048	−0.01	287,055	74.9 ^1^	Positive	+	+
55	Phloridzin	13.73	436.137	436.136	−1.12	435.205, 96.959	-	Positive	+	+
56	Narcissin	13.78	624.169	624.169	0.42	-	-	Positive	+	+
57	Astilbin	14.31	450.116	450.116	−1.03	151.003, 125.023	62 ^1^	Negative	+	+
58	Prunin	14.44	434.121	434.121	0.16	-	-	Positive	+	+
59	Auriculoside	19.28	450.153	450.153	0.74	-	-	Positive	+	+
60	Cirsimarin	19.75	476.132	476.132	0.56	-	-	Positive	+	+
61	Naringin dihydrochalcone	20.16	582.195	582.195	−0.35	-	-	Negative	+	+
62	Phloretin	26.49	274.084	274.084	0.70	-	-	Positive	+	+
63	Nobiletin	27.27	402.131	402.132	0.69	-	-	Positive	+	+
64	Glycitein	27.67	284.068	284.068	−1.86	-	-	Negative	+	+
65	Tiliroside	30.00	594.137	594.137	−0.62	-	-	Positive	+	+
66	Isoliquiritigenin	30.47	256.074	256.073	−0.37	-	-	Positive	+	+
Triterpenoids										
67	Ursolic acid	20.85	456.360	456.360	0.32	203.180, 189.164, 163.148, 95.086	89.6 ^1^/90.2 ^2^	Positive	+	+
68	Lupenone	21.87	424.371	424.371	0.01	-	-	Positive	+	+
69	Oleanolic acid	22.27	456.361	456.361	0.79	-	-	Positive	+	+
70	Cucurbitacin I	25.72	514.293	514.294	1.35	-	-	Positive	+	+
71	Cucurbitacin S	27.39	498.298	498.298	0.38	-	-	Positive	+	+
Fatty acids and derivatives										
72	Suberic acid	11.42	174.089	174.089	−5.92	129.091, 111.080	83.4 ^1^/95.4 ^2^	Negative	+	+
73	Azelaic acid	15.32	188.105	188.104	−5.29	125.096	84.0 ^1^/94.2 ^2^	Negative	+	+
74	Eicosapentaenoic acid	18.11	302.226	302.225	0.99	303.050	-	Positive	+	+
75	3-Oxopalmitic acid	20.62	270.219	270.219	−0.21	-	-	Positive	+	+
76	Adipic acid	21.36	146.058	146.058	0.31	-	-	Positive	+	+
77	Docosahexaenoic acid	22.13	328.240	328.240	−0.18	145.101, 133.106, 119.086,	84.6 ^1^	Positive	+	+
78	(15Z)-9,12,13-Trihydroxy-15-octadecenoic acid	24.10	330.241	330.240	−1.72	329.232, 171.102, 139.112	85.9 ^1^	Negative	+	+
79	Phloionolic acid	29.18	332.256	332.256	−1.15	313.238, 187.097, 157.086	-	Negative	+	+
80	12-Oxophytodienoic acid	29.81	292.204	292.204	0.13	275.204, 147.119, 133.103	84.2	Positive	+	+
81	9,10-Dihydroxystearic acid	33.88	316.261	316.261	−2.14	297.243, 279.233, 171.102, 127.112	-	Positive	+	+
82	13-OH-9Z,11E,15Z-Octadecatrienoic acid	35.43	294.219	294.219	−1.59	275.201, 223.133, 195.138	78.3 ^1^	Negative	+	+
83	13-Hydroxyoctadecadienoic acid	37.34	296.235	296.235	−2.01	277.217, 195.138, 171.102	85.0 ^1^	Negative	+	+
84	16-Hydroxyhexadecanoic acid	43.74	272.235	272.235	−1.75	253.217, 225.222	88.2 ^1^/89.9 ^2^	Negative	+	+
85	Linolenelaidic acid	44.87	278.225	278.225	0.53	123.119, 109.103	-	Positive	+	+
86	Ethyl palmitoleate	48.88	282.256	282.256	−0.12	135.117, 97.102, 83.036, 69.071	79.9 ^1^	Positive	+	+
Amino acids										
87	Histidine	1.55	155.069	155.070	1.21	110.071, 95.061	98.6 ^1^	Positive	+	+
88	Arginine	1.55	174.112	174.112	0.90	130.097, 116.071, 70.061	80.6 ^1^	Positive	+	+
89	Glutamine	1.68	146.069	146.068	−7.42	127.052, 109.034, 84.044	78.7 ^1^/87.8 ^2^	Positive	+	+
90	Adenine	1.75	135.055	135.055	1.65	119.049, 94.066	62.2 ^2^	Positive	+	+
91	Valine	1.77	117.080	117.080	2.63	72.082, 55.055	82.5 ^1^/89.5 ^2^	Positive	+	+
92	Glutamic acid	1.82	147.053	147.053	0.70	-	-	Positive	+	+
93	Proline	1.90	115.063	115.064	3.36	116.071, 70.066	98.7 ^2^	Positive	+	+
94	Leucine	2.60	131.095	131.095	1.34	132.081, 86.097	84.3 ^1^/94.3 ^2^	Positive	+	+
Sugars										
95	α,α-Trehalose	2.34	342.116	342.116	−1.75	161.023, 113.023, 101.023, 89.023	92.5 ^2^	Negative	+	+
Organic acids										
96	Threonic acid	1.74	136.037	136.036	−8.11	135.044, 75.008	84.7^2^	Negative	+	+
97	Gluconic acid	1.74	196.058	196.057	−4.64	177.039, 129.018, 75.008	84.8 ^1^/89.2 ^2^	Negative	+	+
98	*trans*-Aconitic acid	1.77	174.016	174.015	−5.76	129.018, 85.028	62.4 ^1^/88.9 ^2^	Negative	+	+
99	Maleic acid	1.86	116.011	116.010	−9.84	115.002, 71.013	72.7 ^1^/93.3 ^2^	Negative	+	+
100	Malic acid	1.87	134.023	134.020	−8.32	115.002, 71.013	81.0 ^1^/98.7 ^2^	Negative	+	+
101	Succinic acid	2.39	118.027	118.027	2.81	99.008, 73.028	72.4 ^1^/96.0 ^2^	Positive	+	+
102	Citramalic acid	2.31	148.037	148.036	−7.14	129.018, 103.039, 85.028, 59.013	90.5 ^2^	Negative	+	+
103	Propane-1,2,3-tricarboxylic acid	2.35	176.032	176.031	−5.57	157.013, 113.023, 87.020, 69.033	92.7 ^2^	Negative	+	+
104	Citric acid	2.37	192.027	192.026	−4.67	111.008, 87.008, 85.028, 57.033	94.4 ^2^	Negative	+	+
105	Citraconic acid	2.46	130.027	130.025	−8.75	129.018, 85.026	84.8 ^1^/99.9 ^2^	Negative	+	+

Note. t_R_: retention time; L: leaves; Fl: flowers; Score: “-” ChemSpider only; ^1^ mzCloud; ^2^ mzVault; “+”: the substance was detected in the extract.

## Data Availability

Raw data are available upon request.
